# Unsupervised anomaly detection for posteroanterior chest X-rays using multiresolution patch-based self-supervised learning

**DOI:** 10.1038/s41598-023-30589-w

**Published:** 2023-02-28

**Authors:** Minki Kim, Ki-Ryum Moon, Byoung-Dai Lee

**Affiliations:** Division of AI and Computer Engineering, Kyonggi University, Suwon, 16227 Republic of Korea

**Keywords:** Computer science, Information technology

## Abstract

The demand for anomaly detection, which involves the identification of abnormal samples, has continued to increase in various domains. In particular, with increases in the data volume of medical imaging, the demand for automated screening systems has also risen. Consequently, in actual clinical practice, radiologists can focus only on diagnosing patients with abnormal findings. In this study, we propose an unsupervised anomaly detection method for posteroanterior chest X-rays (CXR) using multiresolution patch-based self-supervised learning. The core aspect of our approach is to leverage patch images of different sizes for training and testing to recognize diverse anomalies characterized by unknown shapes and scales. In addition, self-supervised contrastive learning is applied to learn the generalized and robust features of the patches. The performance of the proposed method is evaluated using posteroanterior CXR images from a public dataset for training and testing. The results show that the proposed method is superior to state-of-the-art anomaly detection methods. In addition, unlike single-resolution patch-based methods, the proposed method consistently exhibits a good overall performance regardless of the evaluation criteria used for comparison, thus demonstrating the effectiveness of using multiresolution patch-based features. Overall, the results of this study validate the effectiveness of multiresolution patch-based self-supervised learning for detecting anomalies in CXR images.

## Introduction

Apart from being significantly effective in revealing pathological alterations, chest X-rays (CXRs) possess noninvasive characteristics, exhibit minimal radiation exposure, and are economical. As a result, chest radiography is one of the most frequently performed medical imaging tests for the early diagnosis of numerous lung and heart diseases^1,2^. According to recent surveys on radiography, CXRs account for 40% of the 3.6 billion imaging procedures performed worldwide every year, and this number is expected to continue to increase as more people access medical care^3^. Moreover, as the volume of X-ray data increases, the resultant burden on radiologists increases along with it, as they are compelled to read more studies within a shorter period, which can often lead to diagnostic errors^4^. Thus, a computer system that identifies abnormal CXRs could provide the benefit of not only ensuring the efficient use of radiologists’ time, but also maintaining the quality and safety of radiological care.

Recent advances in deep learning technologies have enabled significant performance improvements in image-anomaly detection. In particular, supervised anomaly detection has demonstrated significantly improved performance by learning from a training dataset containing both normal and abnormal samples^5^. However, implementing supervised approaches for detecting anomalies in CXRs would be ineffective because collecting large-scale labeled anomaly data to fully characterize all notions of anomalousness is often impractical in real clinical settings^6–8^. Instead, since normal CXR images account for an overwhelming proportion of the data, anomaly detection for CXRs can be formulated as an unsupervised one-class classification problem. Essentially, in such a case, anomaly detection learns normality only from normal CXR data, so abnormal CXRs become detectable due to their deviations from the learned model^6^.

Previous studies have reported that subtle radiological findings, such as a small pneumothorax, are more likely to be visible at higher resolutions^9,10^. However, in several cases, deep learning models are trained on reduced low-resolution CXR images owing to the limitations of hardware capabilities, difficulties in training large-scale networks, and significantly increased training time. To address this problem, the input CXR image was split into fixed-size patches, after which the anomaly score for each patch was computed. However, the shortcoming of patch-based approaches is that they may fail to grasp the associations of diverse and unknown scales within an image^11^. Therefore, in this study, we proposed an unsupervised anomaly detection method for CXRs using multiresolution patch-based self-supervised learning. Primarily, our approach involves computing and weighing anomaly scores based on different patch sizes and then aggregating them to generate a final anomaly map that accommodates irregular and variable size anomalies in CXR images. Furthermore, unlike natural images, anomalies in the medical domain tend to strongly resemble normal data^12^. This has created a demand for enhancing the discriminative power of deep features learned from normal CXR data. Therefore, the proposed method employs contrastive learning^13^—a popular form of self-supervised learning—to learn the generalized and robust features of patches.

## Related works

Anomaly detection is a long-studied problem for which a significant number of methods and techniques have been proposed from classical approaches, such as principal component analysis (PCA)^14^, support vector data description (SVDD)^15^, and kernel density estimation^16^, to recent deep learning-based approaches. However, since this study specifically deals with unsupervised anomaly detection for CXR images, our review of related research focuses primarily on methods involving unsupervised deep learning-based anomaly detection in images. Notably, comprehensive surveys on anomaly detection methods can be found in the literature^6–8^.

One such method is the reconstruction-based method^17–22^, which takes a representative approaches to unsupervised image-anomaly detection. The hypothesis adopted by reconstruction-based methods is that models trained only on normal data cannot accurately reconstruct abnormal images. However, this does not always hold true, because a network trained on a single class can successfully represent some out-of-class examples, given that the in-class objects are sufficiently diverse^23^.

Learning the probability distribution of normal data is another representative approach for anomaly detection. The objective of this approach is to model the distribution of normal data directly or to predict whether there are new samples in the distribution^11^. SVDD is a classic one-class classification technique for this category—it essentially fits the smallest possible sphere around the given data points, allowing some points to be excluded as outliers^24^. Furthermore, deep SVDD^25^ improved its predecessor by implementing a deep neural network to minimize the volume of the hypersphere enclosing the feature representations of the data. Moreover, in^26^, vector-quantized variational autoencoder and the autoregressive model were used to estimate the distribution of the latent representation of data. However, methods based on this approach often require careful calibration for learning discriminative data representations^5,11^.

Self-supervised learning (SSL) aims to learn relevant feature representations using pretext tasks. Recently, numerous unsupervised anomaly detection methods have leveraged SSL to learn the discriminative features of normal data that can act as reliable landmarks. For instance, Tan et al.^5^ proposed Poisson image interpolation (PII), which trains a model to detect subtle irregularities introduced via Poisson image editing. For SSL, the PII method produces new training samples by naturally replacing the pixels in a random patch with a convex combination of two normal training samples. Furthermore, patch SVDD^27^ extends deep SVDD for application in a patch-based method using self-supervised learning. Here, the input image is divided into fixed-size patches, each of which is encoded and inspected independently so that a fine-grained examination can be performed. In addition, when used to predict the relative positions of two randomly selected patches, SSL allows the features to form multimodal clusters, thereby enhancing anomaly detection capabilities. Furthermore, Kim et al.^28^ proposed an unsupervised anomaly detection and localization algorithm using a progressive autoencoder combined with patch-level contrastive learning. Instead of applying different transformations to the same patch image, patch images on the same locations of the original image and the restored image by the autoencoder were defined as positive in this study. Unlike conventional contrastive learning approaches, Yoa et al.^29^ leveraged only the positive pairs of normal images to learn global and dense representations. In addition, they extended the learned model using pseudo-abnormal images generated by dynamic local augmentation. Meanwhile, Zhang et al.^30^ proposed a deep adversarial anomaly detection method that utilized SSL along with an adversarial training method for anomaly detection. As an SSL approach, an auxiliary classifier was leveraged to learn task-specific latent features by classifying the original and pseudo data. Hence, the learned features were more discriminative and generic.

The method proposed in this study is similar to patch SVDD^27^, since both utilize patch-based SSL to process highly complex images. However, the proposed methodology has some prominent differences from patch SVDD. First, the rigid partitioning of an image into patches of a fixed size may fail to consider anomalies of different shapes and sizes^11^. Therefore, for a given CXR image, our approach produces separate anomaly maps according to the patch resolutions and then aggregates them to generate the final anomaly map. Second, this study proposes a simple yet efficient algorithm that considers patch locality to compute anomaly scores.

## Materials and methods

Since the current research is a retrospective study, the patients’ informed consent and ethical review for this study were waived by the institutional board of Kyonggi University. All data were obtained from existing and de-identified public datasets. In addition, all methods were performed in accordance with the relevant guidelines and regulations.

### Dataset

PadChest^31^ is a representative large-scale, high-resolution CXR image dataset with multilabel annotated reports that includes over 160,000 images from 67,000 patients labeled with 174 different radiographic findings, 19 different diagnoses, and 104 anatomic locations. In particular, 27% of the reports were manually annotated by radiologists, while the remaining data were annotated automatically using a recurrent neural network with attention mechanisms. From this dataset, we extracted 7,375 manually annotated posteroanterior (PA) CXR images in response to the need to reduce the negative effects of incorrectly labeled data, which consisted of 2574 normal and 4801 abnormal CXR images. The resolution of the CXR images ranged from approximately 1800 × 2100 to 3200 × 3500 pixels with a 12-bit grayscale color depth.

Since the proposed method required only normal CXR images for model training, these images were randomly split into three subsets without overlap: training (70%), validation (15%), and testing (15%). For validation and testing, abnormal CXR images were randomly selected from the 4801 samples without overlap. Table 1 presents the details of the dataset breakdown.

**Table 1:** Dataset details.

	Normal CXR images	Abnormal CXR images
Training	1770	–
Validation	402	402
Testing	402	402

### Proposed method

**Figure 1:**
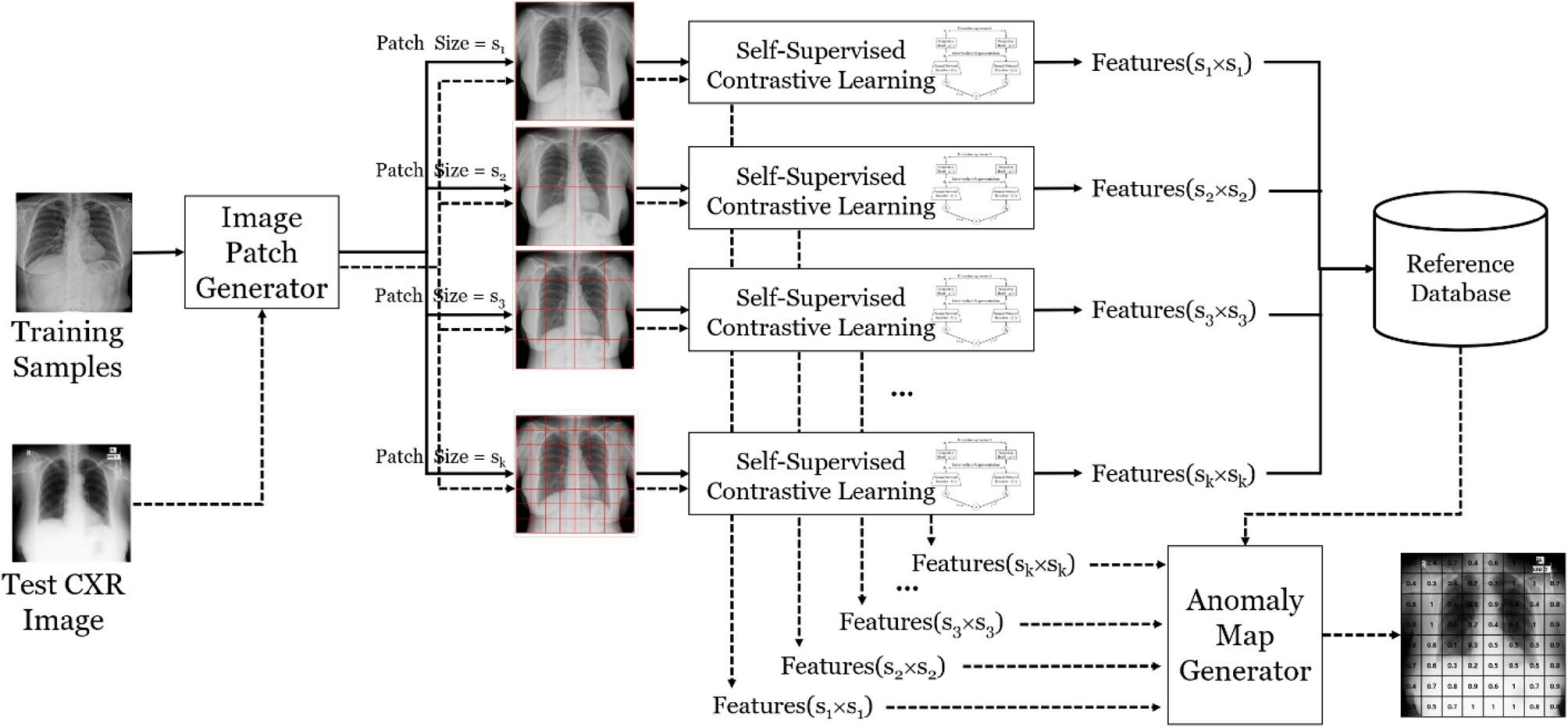
Overall process of anomaly detection in CXR images.

Figure 1 depicts the entire process of detecting abnormal CXR images using the proposed method. The solid and dotted lines represent the training and classification processes, respectively. During the training phase, the CXR images were first split into a group of patch images with a predefined set of patch sizes {*s*_*1*_, *s*_*2*_, …, *s*_*k*_} using the *image patch generator*. The resulting patch images of size *s*_*i*_ were used to train the corresponding deep feature extraction model, D(*s*_*i*_), using self-supervised contrastive learning. After the completion of the training phase, the feature representation of every normal patch image was computed and stored in the reference database as an *n-tuple* containing the patch size, the x and y coordinates of the patch in the original CXR image, and the learned features.

Given a query CXR image x, patch images of different sizes were generated in the same way as in the training process, and their features were extracted from the corresponding deep feature extraction models. Following this, the *anomaly map generator* was used to generate the final anomaly map using the extracted features and the reference database representing the feature space. The anomaly score of each patch image was defined as the inverse of the similarity score to the nearest normal patch in the feature space. Anomaly maps for individual patch sizes were generated using the computed anomaly scores of every patch image. Subsequently, the final anomaly map was generated by aggregating the multiple anomaly maps based on different patch sizes with different weights. The following section presents detailed explanations of the deep feature extraction model and the anomaly map generator.

**Figure 2:**
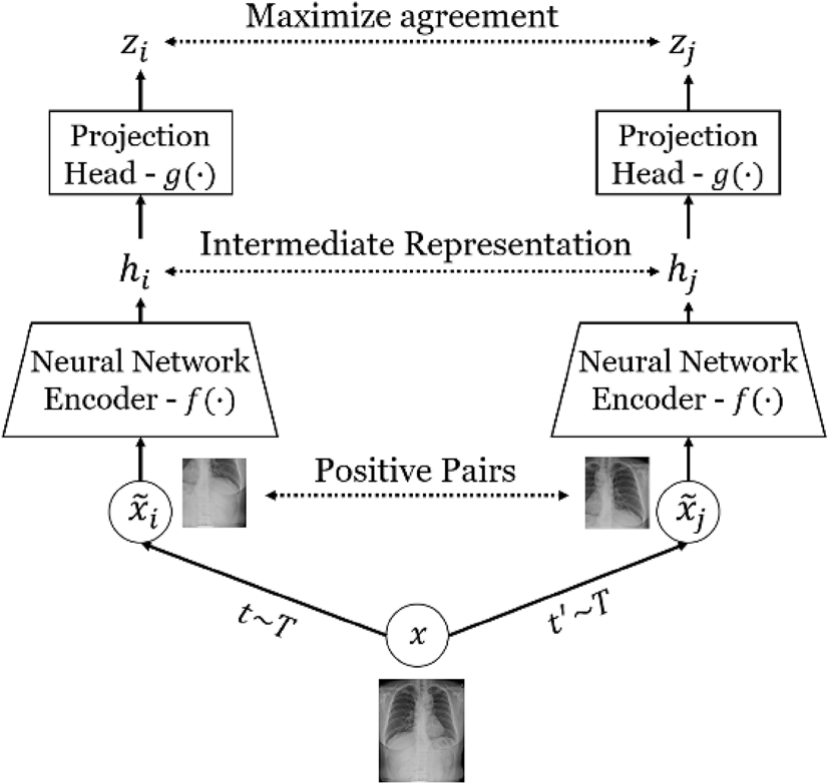
The SimCLR framework^32^. *t* ~ *T* and *t’* ~ *T* are two independent data augmentation operators sampled from the predefined family of augmentations. A pair of samples (${\widetilde{\mathrm{x}}}_{\mathrm{i}},{\widetilde{\mathrm{x}}}_{\mathrm{j}}$) is referred to as positive when generated from the same image. A neural network encoder *f(·)* and projection head *g(·)* are trained to maximize the agreement between the feature representations of the two samples. After completion of the training, the projection head is discarded.

For each patch size *p*, a separate self-supervised deep feature extraction model learns to represent the discriminative features of normal patch images of size *p* using contrastive learning. Recently, contrastive learning has shown promising results in numerous computer vision tasks. In particular, SimCLR is a state-of-the-art self-supervised contrastive learning approach based on contrastive instance discrimination (see Fig. 2) that aims to learn representations by maximizing the agreement between differently augmented views of the same data example using a contrastive loss in the latent space^32^.

The contrastive loss used in SimCLR is defined as the normalized temperature-scaled categorical cross-entropy loss with cosine similarities between representations. Equation (1) presents the loss function for a positive pair of samples (*i*, *j*), where $\sum_{k=1}^{2N}{1}_{[k\ne i]}\in \{0, 1\}$ is an indicator function that returns 1 iff *k* ≠ *i*, (·) denotes the dot product, and τ is a scalar temperature parameter.

1 $${l}_{i,j}=-\mathrm{log}\frac{\mathrm{exp}({z}_{i}\cdot {z}_{j}/\tau )}{\sum_{k=1}^{2N}{1}_{[k\ne i]}\mathrm{exp}({z}_{i}\cdot {z}_{k}/\tau )}.$$

The role of the *anomaly map generator* is to produce anomaly maps based on patch sizes and aggregate them with different weights to produce the final anomaly map. To generate the anomaly map for a given patch size *s*_*i*_, the anomaly map generator first determines the feature representation in the reference database that is most similar to the query patch image of size *s*_*i*_—the higher the similarity score, the more normal the patch image. Therefore, the anomaly score is computed as the inverse of the similarity score. Subsequently, the patch-wise anomaly scores are assigned to the pixels contained in the patches.

**Figure 3:**
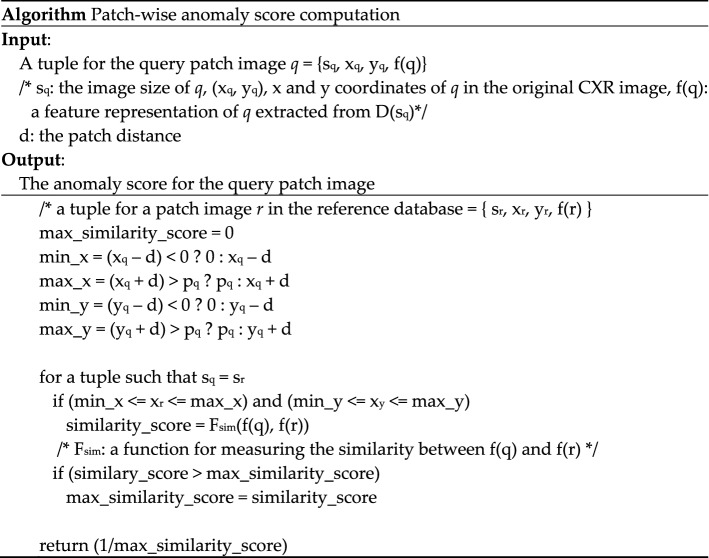
Algorithm for computing the anomaly score for a given query patch image.

**Figure 4:**
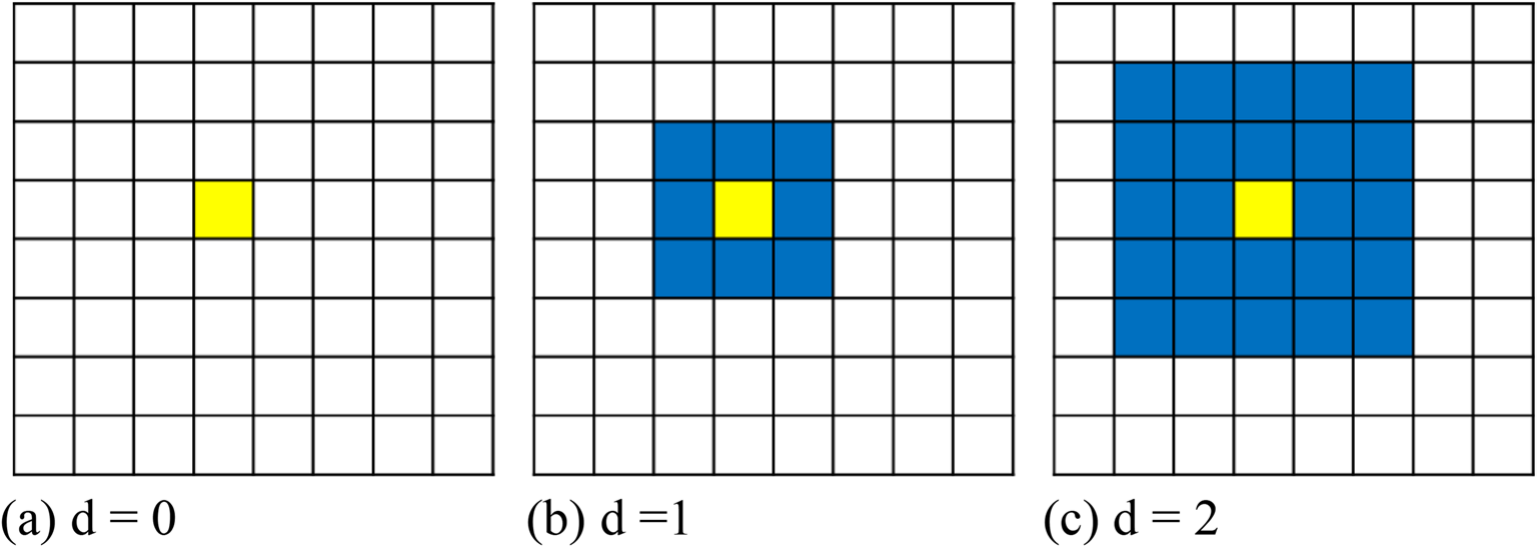
Algorithm for computing the anomaly score for a given query patch image, along with illustrations of the effect of the path distance parameter. The yellow cell indicates the location of a query patch image, and the blue cells refer to the locations of the adjacent reference normal patch images considered in measuring the similarity score with the query patch image, with the reference patch image being at the same location as the query patch image.

Owing to the diversity of organ sizes and shapes and the radiographic positioning of individual patients for CXRs, the same patch positions in two different CXR images may contain dissimilar content. Therefore, simply using the location information of reference normal patches for feature comparison may be inappropriate. However, investigating all the patches in the search space will lead to a significant increase in computational costs. In addition, the feature representations of unrelated patches may be more likely to be returned as search results. Therefore, to minimize the chances of using the feature representations of regions unrelated to the location from which the query patch image was extracted, we used a simple yet efficient algorithm that accounts patch locality. Figure 3 presents the pseudocode for computing the patch-wise anomaly score. In this context, patch distance *d* is a configurable parameter that controls the choice of adjacent patch areas for measuring the similarity score (see Fig. 4).

Given a query patch image, the degree of similarity between *q* and *r* depends on the extent to which *q* follows the data distribution used to train the deep feature extraction model D(*s*_*q*_), and it is measured by the similarity function *F*_*sim*_. In this study, we used several distance functions (e.g., Euclidean distance, Manhattan distance, cosine similarity, and Mahalanobis distance) as similarity functions to examine each of their performances. For two given feature representations, X = (*x*_*1*_, *x*_*2*_, …, *x*_*n*_) and Y = (*y*_*1*_, *y*_*2*_, …, *y*_*n*_), the corresponding equations for the individual distance functions are shown in (2)–(5), where *S* denotes the sample covariance matrix.

2 $${D}_{euclidean}=\sqrt{\sum_{i=1}^{n}{({x}_{i}-{y}_{i})}^{2}},$$

3 $${D}_{manhattan}=\sum_{i=1}^{n}\left|{x}_{i}-{y}_{i}\right|,$$

4 $${D}_{cosine\_sim}=\frac{p\cdot q}{\Vert p\Vert \Vert q\Vert }=\frac{\sqrt{\sum_{i=1}^{n}{x}_{i}\times {y}_{i}}}{\sqrt{\sum_{i=1}^{n}{({x}_{i})}^{2}}\sqrt{\sum_{i=1}^{n}{({y}_{i})}^{2}}},$$

5 $${D}_{mahalanobis}=\sqrt{{\left(X-Y\right)}^{T}{S}^{-1}(X-Y)}.$$

Once the anomaly maps were generated according to the patch sizes (e.g., *A(s*_*i*_*)*), the final anomaly map (e.g., *A*_*final*_) was produced by aggregating the anomaly maps using element-wise multiplication with weights and then normalizing the resulting intermediate anomaly map, as shown in (6), where *w*_*i*_ is the weight assigned to the anomaly map for a patch image of size *s*_*i*_.

6 $${A}_{final}={Normalize(w}_{1}A\left({s}_{1}\right)\odot {w}_{2}A\left({s}_{2}\right)\odot \dots \odot {w}_{k}A({s}_{k})).$$

Following this, determining whether the given query CXR image is abnormal was quite simple. The anomaly score of the query CXR image was represented by the highest anomaly score of the pixels in *A*_*final*_—the CXR image was considered abnormal if its anomaly score was larger than the predefined threshold, while the pixels with higher anomaly score values approximated the abnormal regions.

## Experimental results and discussion

### Evaluation metrics and training details

The performance of the proposed method was evaluated in terms of its accuracy, precision, recall, and specificity. The corresponding equations for the evaluation metrics are noted in (7)–(10), where TP, FP, TN, and FN indicate true positive, false positive, true negative, and false negative predictions, respectively. In addition, the area under the receiver operating characteristics (AUROC) curve was measured to evaluate the accuracy of the classification performance.

7 $$Accuracy=\frac{\mathrm{TP}+\mathrm{TN}}{\mathrm{TP}+\mathrm{TN}+\mathrm{FP}+\mathrm{FN}}.$$

8 $$Precision=\frac{\mathrm{TP}}{\mathrm{TP}+\mathrm{FP}},$$

9 $$Sensitivity=\frac{\mathrm{TP}}{\mathrm{TP}+\mathrm{FN}},$$

10 $$Specificity=\frac{\mathrm{TN}}{\mathrm{TN}+\mathrm{FP}}.$$

For the final evaluation, we applied five-fold cross-validation to the dataset; for each fold, we measured the corresponding performance metrics, and the final result was the averaged result.

Furthermore, as our contrastive learning model, we used SimCLR^32^ and trained ResNet-50 as the base encoder network and a two-layer MLP projection head using four NVIDIA GeForce RTX 2080ti graphic cards in an Ubuntu 18.04.5 LTS environment. The encoder network produced $h=f(x)\in {R}^{2048}$ features, while the projection head produced $z=g(h)\in {R}^{128}$ features. The mini-batch size and the number of training epochs were set to 256 and 100, respectively. For data augmentation, we used random cropping (50%) and rotation (from – 5° to + 5°) to process the grayscale CXR images. The entire network was trained in an end-to-end manner using a LARS optimizer with a learning rate of 4.8 and weight decay of 10^−6^, as in the SimCLR^32^. Prior to training and evaluation, the CXR images were resized to 1024 × 1024 pixels without normalization. In addition, since a patch with a resolution of 128 × 128 pixels was considered as the minimum size containing distinguishable features in the CXR images, the predefined set of patch resolutions was set to {128, 256, 512, 1024}. Throughout the experiments, the patch distance parameters for the 128 × 128 and 256 × 256 patch images were set to 2 and 1, respectively. For other patch resolutions (e.g., 512 × 512 and 1024 × 1024 pixels), the patch distance parameters were set to 0. The threshold for determining abnormality was set to 0.5. Note that the objective of this study was not to determine the optimal parameter settings, and the search for the optimal hyperparameters values is left for future studies.

### Results and discussion

**Figure 5:**
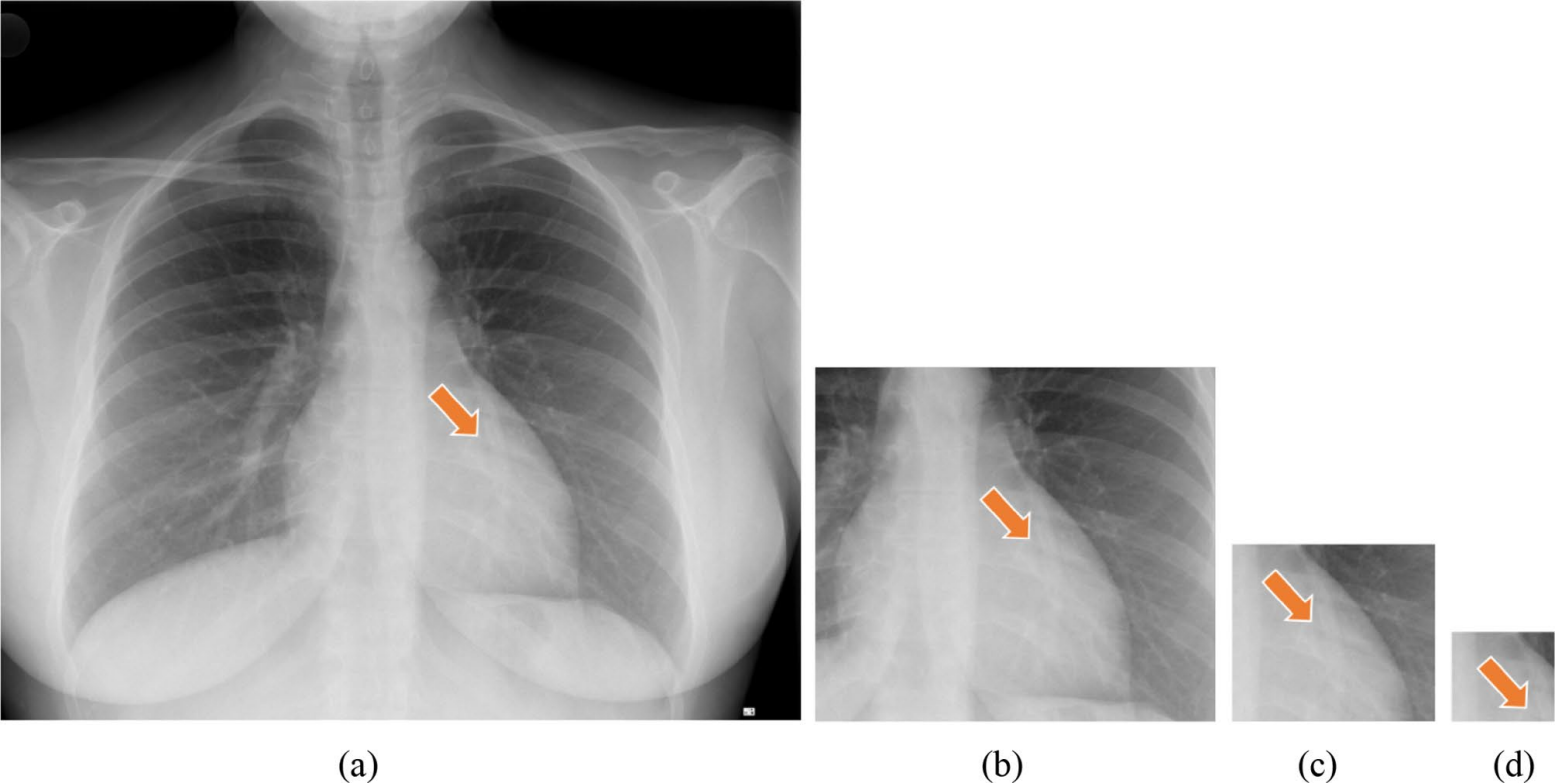
(**a**) Effect of patch resolution on identifying radiographic findings. An input CXR image of size W × H is divided into patch images of different sizes. (**b**–**d**) Examples of resulting patch images of sizes (**b**) W/2 × H/2, (**c**) W/4 × H/4, and (**d**) W/8 × H/8. The arrows identify the location of the patch images within the original input CXR image.

We first conducted an experiment to assess the effectiveness of the multiresolution patch-based anomaly detection method over single-resolution patch-based methods. For this purpose, the proposed method was modified to use patch images of a fixed size for both the training and testing phases. Other components, including the SimCLR, were applied without changes. For each single-resolution patch-based method, we evaluated their performance according to the similarity function used. Table 2 lists the quantitative results, where the values in bold denote the best performances. The results indicate that the patch resolution providing the best performance varied depending on the evaluation criteria. For instance, the combination of 1024 × 1024 patch resolution with Euclidean distance function as the similarity function outperformed the other settings in terms of sensitivity (e.g., 0.71), whereas the combination of the patch resolution of 512 × 512 with Mahalanobis distance exhibited the best performance (0.74) for the AUROC. In general, methods using higher patch resolutions for each similarity function tended to provide better overall performances. These phenomena can be explained by Fig. 5, which presents patch images of different resolutions for a given CXR image. Herein, the patch size may be regarded as the receptive field. Therefore, although small patch sizes are known to be helpful in capturing local contexts, they lack the ability to gather global semantic information that are critical for determining structural deformities. For instance, approximating the left cardiac shape becomes difficult as the patch size decreases.

**Table 2:** Comparison of the performances of single-resolution patch-based anomaly detection.

Similarity function	Patch size	Accuracy	Precision	Sensitivity	Specificity	AUROC
*D*_*euclidean*_	128 × 128	0.58	0.61	0.43	0.51	0.62
	256 × 256	0.62	0.60	0.69	0.51	0.64
	512 × 512	0.61	0.60	0.67	0.55	0.63
	1024 × 1024	0.60	0.58	**0.71**	0.53	0.63
*D*_*manhattan*_	128 × 128	0.54	0.55	0.47	0.61	0.56
	256 × 256	0.60	0.61	0.55	0.63	0.62
	512 × 512	0.61	0.62	0.55	0.67	0.63
	1024 × 1024	0.60	0.61	0.55	0.67	0.62
*D*_*cosine_sim*_	128 × 128	0.48	0.48	0.40	0.56	0.50
	256 × 256	0.51	0.49	0.43	0.55	0.52
	512 × 512	0.65	0.68	0.57	0.73	0.67
	1024 × 1024	0.65	0.68	0.58	0.74	0.68
*D*_*mahalanobis*_	128 × 128	0.60	0.72	0.55	0.79	0.56
	256 × 256	0.71	**0.82**	0.54	**0.88**	0.72
	512 × 512	**0.72**	0.75	0.45	0.85	**0.74**
	1024 × 1024	0.69	0.75	0.57	0.86	0.73

Similarly, we evaluated the multiresolution patch-based anomaly detection method by changing the weights of the patch sizes used to generate the anomaly map and the similarity function. Table 3 presents a subset of the results that exhibited relatively good performances. The terms w1, w2, w3, and w4 represent the weights of the patch images of sizes 128 × 128, 256 × 256, 512 × 512, and 1024 × 1024, respectively. Similar to the results presented in Table 3, features extracted from the patch images of size 128 × 128 had a relatively low impact in determining abnormalities in the CXR images; thus, configurations exhibiting good performance had considerably low values for the corresponding weight (e.g., w1). The results clearly demonstrate that fusing the features of patch images of different sizes plays a crucial role in constructing discriminative features for anomaly detection. In particular, the multiresolution patch-based anomaly detection method, with the Mahalanobis distance and w1, w2, w3, and w4 set to 0.1, 1.2, 1.2, and 1.0, respectively, exhibited comparable or better performance than the best performances obtained by single-resolution patch-based methods, regardless of the evaluation criteria. Meanwhile, although the single-resolution patch-based methods exhibited different performances depending on the evaluation criteria, the multiresolution patch-based method with the selected configuration [e.g., Mahalanobis distance, weights = (0.1, 1.2, 1.2, 1.0)] consistently exhibited good overall performances, regardless of the evaluation criteria used for the comparisons.

**Table 3:** Comparison of the performances of multi-resolution patch-based anomaly detection methods.

Similarity function	Weights (w1, w2, w3, w4)	Accuracy	Precision	Sensitivity	Specificity	AUROC
*D*_*cosine_sim*_	(1.0, 1.0, 1.0, 1.0)	0.62	0.68	0.53	0.76	0.66
	(1.0, 1.2, 1.2, 1.2)	0.63	0.69	0.54	0.75	0.66
	(0.1, 1.0, 1.0, 1.0)	0.67	0.72	0.55	0.79	0.69
	(0.1, 1.2, 1.0, 1.0)	0.64	0.68	0.54	0.74	0.67
	(0.1, 1.2, 1.2, 1.0)	0.65	0.68	0.54	0.74	0.67
	(0.1, 1,0, 1.0, 1.2)	0.66	0.68	0.54	0.74	0.67
	(0.1, 1.0, 1.4, 1.0)	0.65	0.69	0.54	0.76	0.67
*D*_*mahalanobis*_	(1.0, 1.0, 1.0, 1.0)	0.68	0.75	0.57	0.83	0.70
	(1.0, 1.2, 1.2, 1.2)	0.69	0.77	0.57	0.82	0.69
	(0.1, 1.0, 1.0, 1.0)	0.72	0.81	0.58	0.85	0.73
	(0.1, 1.2, 1.0, 1.0)	0.72	0.81	0.58	0.85	0.73
	(0.1, 1.2, 1.2, 1.0)	0.73	0.83	0.59	0.89	0.75
	(0.1, 1,0, 1.0, 1.2)	0.71	0.78	0.59	0.83	0.73
	(0.1, 1.0, 1.4, 1.0)	0.71	0.78	0.58	0.84	0.72

Additionally, we compared the proposed method with recent state-of-the-art unsupervised anomaly detection methods: f-AnoGAN^20^, patch SVDD^27^, Pix2PixHD^33^, Salehi et al.^34^, and SQUID^35^. The f-AnoGAN is a representative reconstruction-based anomaly detection method in which two adversarial networks (generator and discriminator) and an encoder are separately trained using normal images to map input images to the corresponding locations of the learned latent representation. For this experiment, we modified the sizes of the input and patch images of the official f-AnoGAN implementation to process the CXR images. In particular, the first version, f-AnoGAN(1), resized an input CXR image into 512 × 512 pixels and extracted 2D image patches of 128 × 128 pixels at randomly sampled positions from the input image. The second version, f-AnoGAN(2), used the same input image size but used 256 × 256 pixels as the patch image size. Similarly, we modified the official implementation of the patch SVDD to use an input size of 256 × 256 pixels and a patch size of 64 × 64 pixels, denoted as Patch SVDD(1). For another version, denoted as Patch SVDD(2), we used input image and patch sizes of 1024 × 1024 and 256 × 256 pixels, respectively. Note that the selection of the image and patch sizes accounts for the constraints of the computational resources used in the experiments. For comparison, we included a deep autoencoder-based anomaly detection method^7^, which has been frequently adopted as a baseline performance indicator. In particular, we replaced the symmetric encoder-decoder architecture of a deep autoencoder with Pix2PixHD^33^ to process high-resolution CXR images. For the Pix2PixHD-based anomaly detection system, the input CXR images were resized to 1024 × 1024 pixels, and a latent vector of size 1 × 256 × 256 was used for feature representation. For Salehi et al.^34^ and SQUID^35^, we used their official implementations and hyperparameter settings, which were reported to have delivered the best performances in their original publications. In addition, all CXR images were resized to 256 × 256 and 128 × 128 pixels as inputs for Salehi et al. and SQUID, respectively, consistent with the values used in the publications. Meanwhile, for the multiresolution patch-based method, we used the Mahalanobis distance as the similarity function and set w1, w2, w3, and w4 to 0.1, 1.2, 1.2, and 1.0, respectively, which exhibited the best performance (Table 3).

Table 4 lists the performances of all the methods investigated in the comparison conducted in this study. For the f-AnoGAN methods, using different patch sizes did not affect the overall performance of either method. In contrast, patch SVDD(2) used input image and patch sizes that were four times larger than patch SVDD(1), thus outperforming patch SVDD(1) by a large margin (> 8.9%) in all aspects. This result can certainly be attributed to image size. High-resolution images enabled patch SVDD(2) to learn to extract fine feature representations to enable subtle radiological findings. In addition, the encoder can benefit from a larger patch size since an enlarged receptive field enables the feature representation to explicitly learn long-range dependency. Furthermore, the performance of the Pix2PixHD-based anomaly detection method was slightly better than that of the patch SVDD(2). We conjecture that the Pix2PixHD-based anomaly detection method suffered less from the limited receptive field and could capture long-range dependencies because the Pix2PixHD-based anomaly detection method learned latent features for normal data without patches. Interestingly, despite using smaller CXR images, both Salehi et al. and SQUID achieved competitive or better performances than the Pix2PixHD-based method. These results may be attributed to their use of more sophisticated algorithms and techniques for modeling the distribution of normal features, such as image in-painting-based data augmentation and multiresolution knowledge distillation. Among all methods, the proposed method was observed to deliver better performances with regard to all evaluation criteria except sensitivity. For instance, the proposed method achieved accuracy, precision, specificity, and AUROC of 0.73, 0.83, 0.89, and 0.75, with improvements of 8.96%, 27.69%, 43.55%, and 4.17%, respectively, compared to Salehi et al.^34^ which showed the best performance in the comparison group.

**Table 4:** Comparison of the performances of the proposed method with state-of-the-art methods for anomaly detection in CXR images.

Models	Accuracy	Precision	Sensitivity	Specificity	AUROC
f-AnoGAN(1)	0.26	0.27	0.28	0.25	0.50
f-AnoGAN(2)	0.26	0.26	0.25	0.28	0.50
Patch SVDD(1)	0.32	0.34	0.37	0.28	0.56
Patch SVDD(2)	0.52	0.52	0.47	0.57	0.61
Pix2PixHD-based	0.58	0.59	0.54	0.62	0.66
Salehi et al.^34^	0.67	0.65	0.72	0.62	0.72
SQUID^35^	0.57	0.61	0.40	0.74	0.58
Ours	0.73	0.83	0.59	0.89	0.75

**Figure 6:**
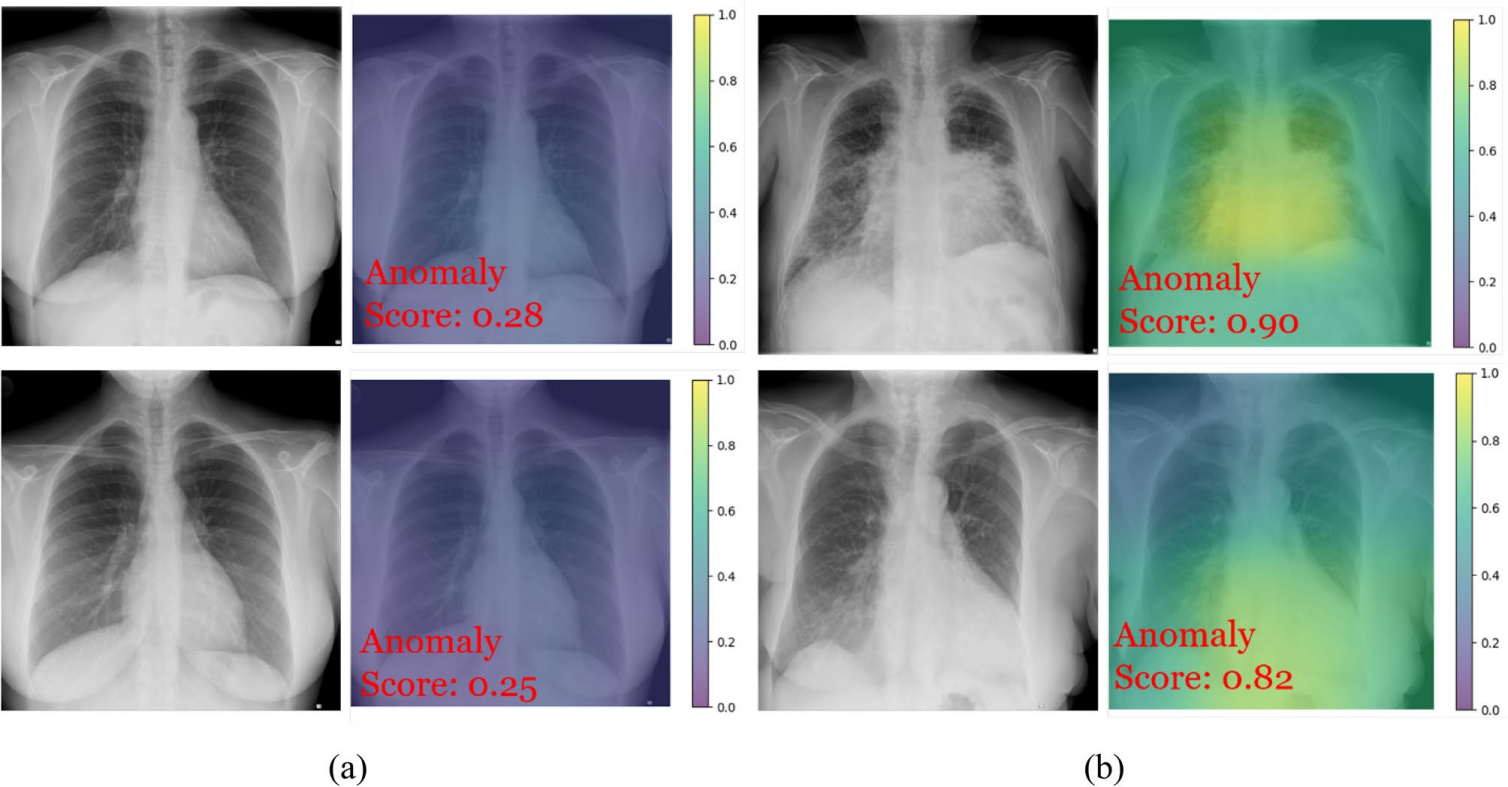
Radiographs of selected correctly classified examples—the anomaly scores were generated by the proposed method that showed the best performance in the experiments: (**a**) Truth label: normal, predicted label: normal; (**b**) truth label: abnormal, predicted label: abnormal.

Figure 6 illustrates the test examples of CXR images and their corresponding anomaly maps that are correctly classified. For the normal test CXR images, the proposed method found the nearest normal patch images in the learned feature space and confirmed that the corresponding query CXR images contained no anomalies. For the abnormal test CXR images, the top one is a PA CXR of a patient with alveolar pattern and the bottom one is a PA CXR of a patient with cardiomegaly. These examples confirm that the deep features of normal patches were learned to be separable from abnormal ones using the proposed method. Although our method was able to determine CXR images as normal or anomalous while exhibiting a competitive performance, there is still room for improvement in anomaly localization, as shown in Fig. 6. Anomaly localization requires accurate computation of pixel-level anomaly scores. In our model, however, patch-wise calculated anomaly scores were assigned to all the pixels within a patch, which made it difficult to disassociate anomalous pixels from normal ones. In addition, unsupervised learning without ever seeing the anomalous samples of any class during training makes this task even more challenging.

**Figure 7:**
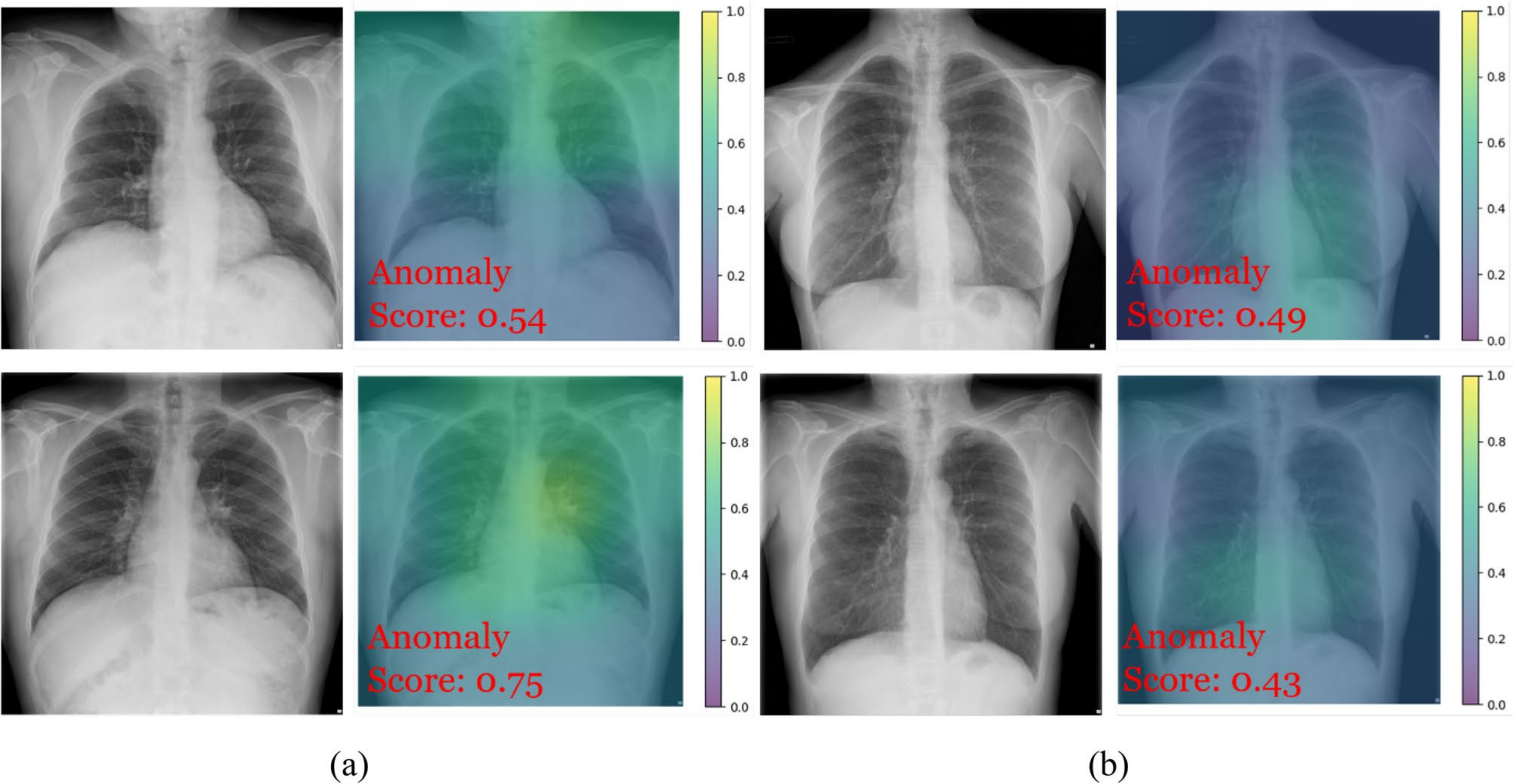
Radiographs of selected misclassified examples—the anomaly scores were generated by the proposed method that showed the best performance in the experiments: (**a**) Truth label: normal, predicted label: abnormal; (**b**) truth label: abnormal, predicted label: normal.

Figure 7 depicts the radiographs of selected misclassified examples. According to the anomaly score of the top CXR image in Fig. 7a, the extracted latent features for the query CXR image resided in the boundary area of the feature space between normal and abnormal, making accurate prediction difficult. The bottom CXR image in Fig. 7b is an example in which the proposed method misclassified the presence of anomalies, such as a lung mass. These limitations can be addressed by adding more normal CXR images when training the encoder for feature representation. However, despite their strong visual similarities, two CXR images may be classified differently due to their subtle differences, which is an inherent challenge in medical anomaly detection. For instance, the CXR images in Fig. 7b are those of patients with pleural thickening (top image in Fig. 7b) and air trapping (bottom image in Fig. 7b). To address such challenging cases and improve the generalization capability of deep-learning-based approaches for anomaly detection, more sophisticated algorithms and methods for feature representation are required, along with additional large-scale training datasets.

## Conclusions

This study proposed an unsupervised anomaly detection method for CXR images using multiresolution, patch-based, self-supervised learning. The proposed method leveraged patch images of different resolutions to address problems related to the rigid partitioning of input images to patches of a fixed size. Furthermore, self-supervised contrastive learning was applied to train the feature extraction models for patch images, thereby producing discriminative deep feature representations. Related experiments were conducted on a public dataset to assess the validity of the proposed method. The results show that, unlike single-resolution patch-based methods, the proposed method performs consistently well regardless of the evaluation criteria, thus demonstrating the effectiveness of utilizing multiresolution patch-based features. Furthermore, the proposed method significantly outperformed other state-of-the-art anomaly detection methods.

Future research in this area of study should investigate several pertinent aspects. First, we plan to study the behavior of the proposed method by changing its core components, such as image partitioning strategies and contrastive learning algorithms. Second, we intend to apply the semi-supervised approach to the proposed method and use labeled abnormal data to improve performance. Such in-depth investigations into anomaly detection in CXR could contribute to further improving and refining the proposed method, which will have positive implications for radiology as a whole.

## Data Availability

The public dataset used in this study is accessible at http://bimcv.cipf.es/bimcv-projects/padchest/ (accessed on October 5, 2022).
